# The pharmacological role of histone demethylase JMJD3 inhibitor GSK-J4 on glioma cells

**DOI:** 10.18632/oncotarget.19793

**Published:** 2017-08-02

**Authors:** Aixia Sui, Yongbing Xu, Yitong Li, Qilu Hu, Zeyang Wang, Hongtao Zhang, Junjie Yang, Xiaoqiang Guo, Wenqing Zhao

**Affiliations:** ^1^ Faculty of Graduate Studies, Hebei Medical University, Shijiazhuang 050081, Hebei, China; ^2^ Department of Oncology, Hebei General Hospital, Shijiazhuang 050051, Hebei, China; ^3^ Department of Neurosurgery, Hebei General Hospital, Shijiazhuang 050051, Hebei, China; ^4^ State Engineering Laboratory of Medical Key Technologies Application of Synthetic Biology, Key Laboratory of Medical Reprogramming Technology, Shenzhen Second People's Hospital, The First Affliated Hospital of Shenzhen University, Shenzhen 518035, Guangdong, China; ^5^ Department of Urology, Peking University Shenzhen Hospital, Institute of Urology of Shenzhen PKU-HKUST Medical Center, Shenzhen 518036, Guangdong, China

**Keywords:** glioma, histone demethylase, JMJD3, inhibitor, GSK-J4

## Abstract

Glioma is regarded as the most prevalent malignant carcinoma of the central nervous system, and lack of effective treatment. Thus, the development of new therapeutic strategies targeting glioma is of significant clinical importance. In the present study, histone H3K27 demethylase jumonji domain-containing protein 3 (JMJD3) was investigated as target for glioma treatment. The mRNA of JMJD3 was overexpressed in glioblastoma tissues compared to normal brain tissues (*P*<0.05). The content of JMJD3 was also higher in glioma cells than in human brain microvascular endothelial cell (hCMEC), and the corresponding level of H3K27me3 was decreased (*P*<0.05). The treatment with JMJD3 specific inhibitor GSK-J4 can increase the content of H3K27me3 in glioma cells, which means the activity of JMJD3 was inhibited. GSK-J4 can inhibit glioma cell proliferation in a concentration dependent and time-dependent manner (*P*<0.05). GSK-J4 also induced glioma cell apoptosis and inhibited cell migration (*P*<0.05). But there was no obvious effect of GSK-J4 on hCMEC cells. All together, these data suggest that GSK-J4 has important potential in the gliomas treatment.

## INTRODUCTION

Brain glioma is one of the most common malignant brain tumors, accounting for about 40% of the total brain tumors and 80% of all malignant brain tumors [1]. Glioma patients were treated mainly with surgery, radiotherapy and chemotherapy, most of which has a poor prognosis [2]. So, it is of great significance to explore new therapeutic strategies for glioma. A series of research advances on glioma mechanism provide numerous therapeutic targets [3].

Epigenetic abnormity is an important factor of gliomas development [4]. The studies on paediatric glioblastoma provide new clues for gliomas development [5]. In paediatric glioblastoma multiforme, there are 31% mutations in *H3F3A* gene which codes histone 3 variant H3.3 [6]. The commonest *H3F3A* mutations lead to amino acid substitutions at lysine (K) 27 to methionine (M) (K27M) [7]. The K27M mutation can reduce H3K27me3 levels and activate gene expression [8], which is considered as main cause of K27M mutation related tumorigenesis.

The inhibition of histone demethylation can be an effective strategy for gliomas treatment [9]. Jumonji domain-containing protein 3 (JMJD3) specific inhibitor GSK-J4 can increase H3K27 methylation in K27M mutated gliomas, and has antitumor activity against K27M cells and K27M xenografts [10]. Combination GSK-J4 and deacetylase inhibitor panobinostat had synergistic effects on K27M gliomas treatment [11]. But, it is not clear whether JMJD3 inhibition is suitable for the treatment of non-K27M mutated glioma.

In this study, we demonstrated that histone H3K27 demethylase JMJD3 is overexpressed in gliomas tissues, and also higher in glioma cells than endothelial cells. While, corresponding H3K27me3 content is lower in glioma cells. JMJD3 inhibitor GSK-J4 can inhibit cell proliferation and migration, and promote cell apoptosis in glioma cells. But the effect is not obvious in endothelial cells. These results suggested that GSK-J4 can not only play anti-cancer activity on K27M mutated glioma cell, but also be effective on a broader spectrum of glioma.

## RESULTS

### The mRNA of JMJD3 is up-regulated in glioblastoma

**Figure 1:**
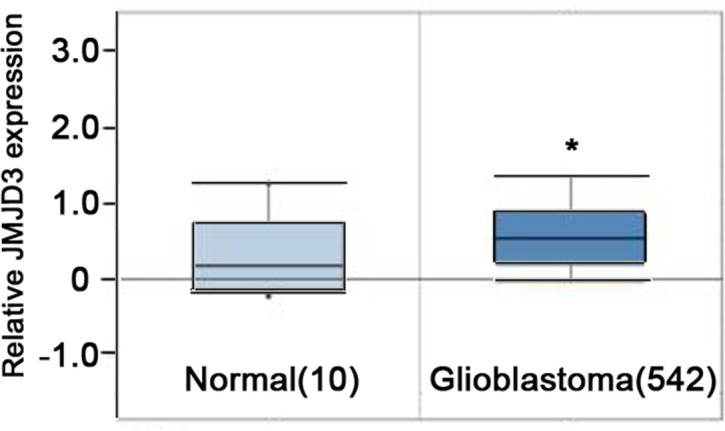
*JMJD3* is overexpressed in brain glioblastoma Oncomine data mining analysis of *JMJD3* mRNA levels between normal brain tissues versus glioblastoma tissues. *, *P* < 0.05.

To determine the value of the JMJD3 intervention in the treatment of glioma, we first examined the expression of JMJD3. Taken similar strategies with Chen et al [12], we performed data mining and analyzed JMJD3 expressions from the publicly available Oncomine database. In the database, JMJD3 was obviously up-regulated in tumor tissues of glioblastoma compared with normal brain tissues (*P*<0.05, Figure 1). These results suggested that targeting JMJD3 has potential clinical value in the glioma treatment.

### The JMJD3 is overexpressed in glioma cells

**Figure 2:**
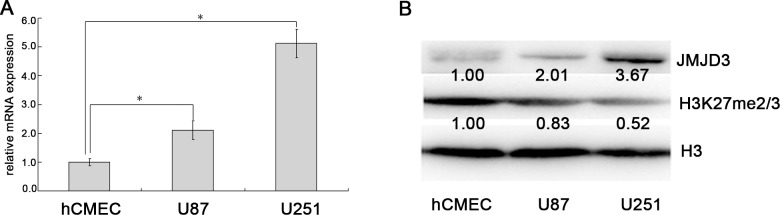
*JMJD3* is overexpressed in glioma cells **(A)** *JMJD3* mRNA expression was higher in glioma cells U87 and U251 than endothelial cells hCMEC. **(B)** The content of JMJD3 protein was higher in U87 and U251 than hCMEC, and the corresponding H3K27me3 level was lower. *, *P* < 0.05.

Then, we measured the expression of JMJD3 in glioma cells and control endothelial cells. These results indicated that levels of JMJD3 mRNA and protein were obviously increased in glioma cells U87 and U251 compared to hCMEC (Figure 2A and 2B), while the content of H3K27me3 was reduced (Figure 2B). This result implied that both glioma cell lines can be used as JMJD3-positive glioma models to further carry out *in vitro* experiments.

### GSK-J4 reduces H3K27me3 content

**Figure 3:**
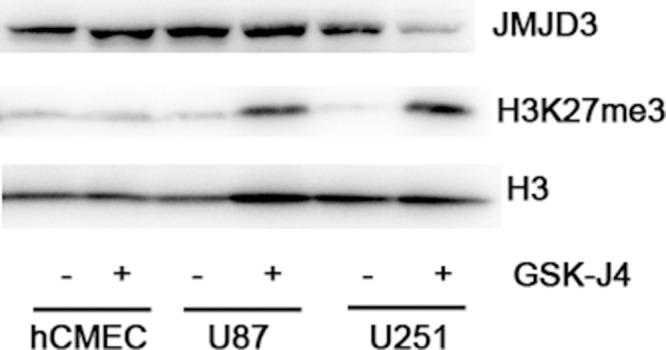
GSK-J4 decreases the content of H3K27me3 in glioma cells U87, U251 and hCMEC cells were treated with 4μM GSK-J4 for 24h, and the H3K27me3 levels were determined with western blotting. Total H3 served as a loading control.

In order to determine the biological activity of GSK-J4, the content of H3K27me3 was measured with western blotting after cell treatment with GSK-J4. These results indicated GSK-J4 can obviously increase the content of H3K27me3 in glioma cell lines U87 and U251 (Figure 3), but no significant effect on hCMEC. This result illustrated that GSKJ4 can effectively inhibit the enzymatic activity of JMJD3 in glioma cells.

### GSK-J4 inhibits the cell proliferation of glioma cells

**Figure 4:**
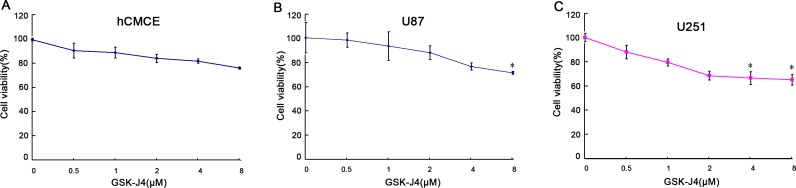
GSK-J4 inhibits the proliferation of glioma cells in a concentration dependent manner hCMEC **(A)**, U87 **(B)** and U251 **(C)** cells were treated with GSK-J4 at indicated concentration for 24h, and the effects on cell proliferation were determined with CCK-8 assay. *, *P* < 0.05.

**Figure 5:**
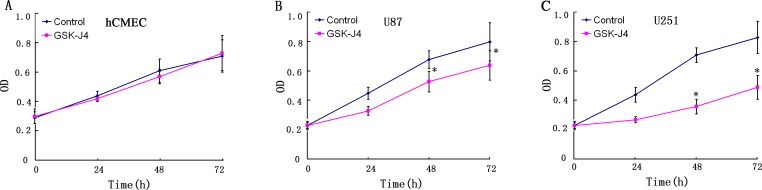
The inhibitory effects of GSK-J4 glioma cells in a time dependent manner Three cells hCMEC **(A)**, U87 **(B)** and U251 **(C)** were treated with 4μM GSK-J4 for 24h, 48h and 72h, and the effects on cell proliferation were determined with CCK-8 assay.*, *P* < 0.05.

To understand the effect of GSK-J4 on cell proliferation of glioma cells, CCK8 assay was followed. The cell proliferation was significantly inhibited in U87 and U251 cells after GSK-J4 treatment in a concentration dependent and time-dependent manner (*P*<0.05, Figures 4 and 5). But this is no obviously inhibition of GSK-J4 on hCMEC. These results indicated that GSKJ4 has anti-proliferative effect on glioma cells.

### GSK-J4 promotes cell apoptosis of glioma cells

**Figure 6:**
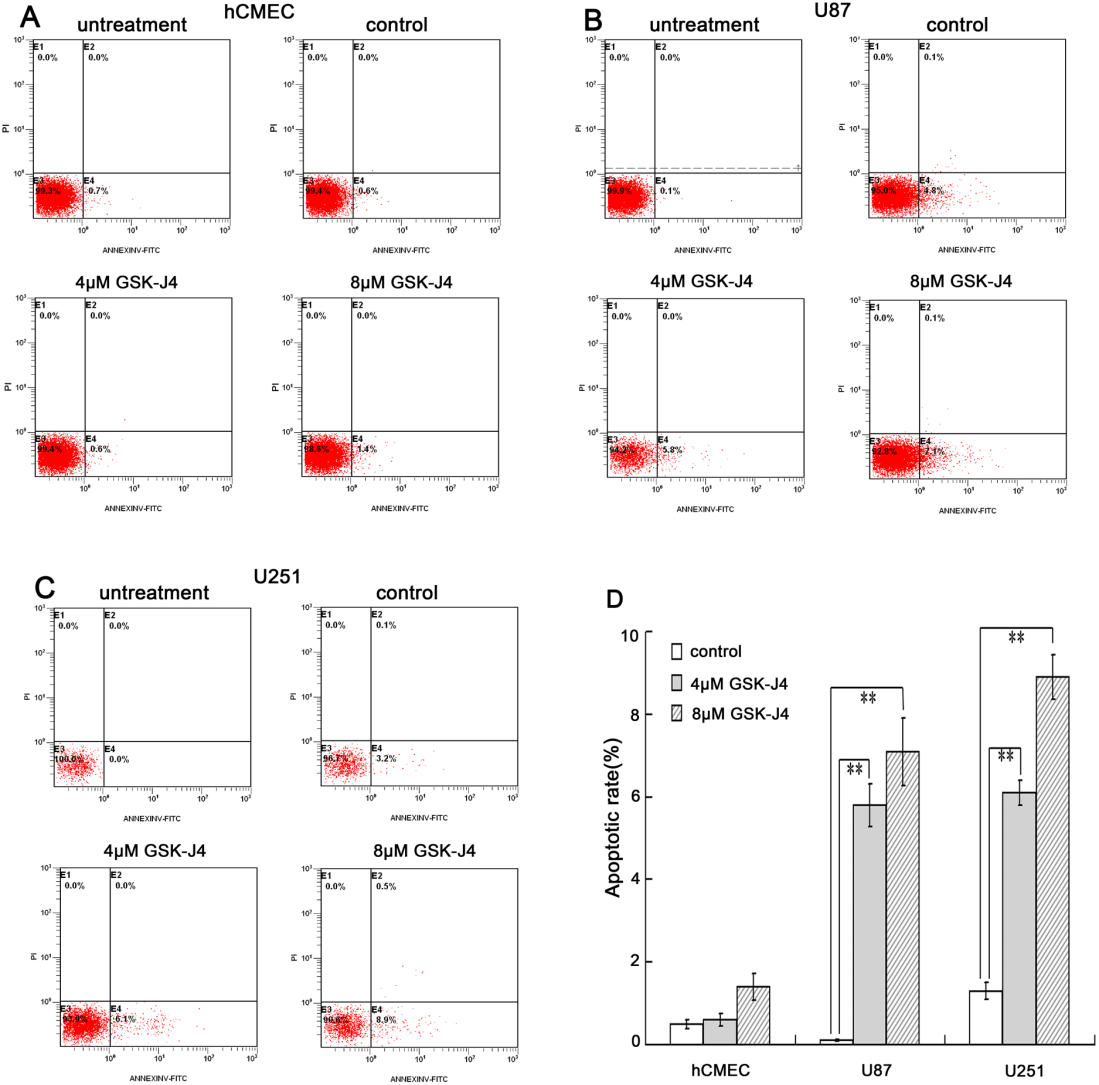
GSK-J4 promotes the apoptosis of glioma cells The three cell lines were treated with 4μM or 8μM GSK-J4 for 24h, and the cell apoptosis changes were determined by flow cytometry analysis. **(A)** hCMEC, **(B)** U87, **(C)** U251, **(D)** quantitative results of cell apoptosis. **, *P* < 0.01.

To explore the role of GSK-J4 on cell apoptosis, the flow cytometry analysis was performed. The data indicated that GSK-J4 can significantly induce cell apoptosis in glioma cells U87 and U251 (*P*<0.05, Figure 6B, 6C and 6D). On the contrary, there was no difference in apoptotic rate in hCMEC cell after GSK-J4 treatment (Figure 6A and 6D). All these results showed that GSK-J4 can selectively inhibit glioma cell proliferation and induce glioma cell apoptosis, and also implied that GSK-J4 has fewer side effects on normal cells.

### GSK-J4 inhibits cell migration of glioma cells

**Figure 7:**
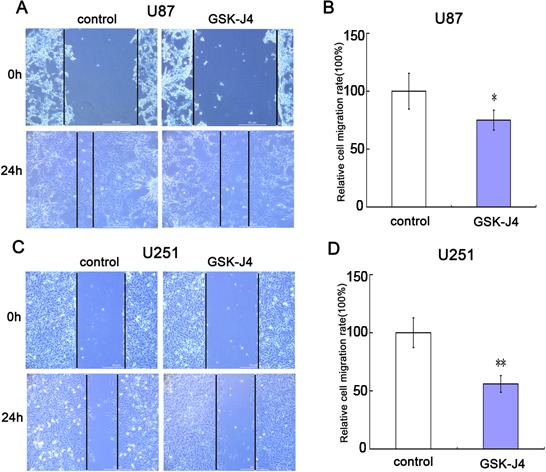
GSK-J4 inhibits cell migration of glioma cells Both U87 and U251 cells were treated with 8μM GSK-J4 for 24h, and the effects on cell migration were determined with cell scratch test. **(A)** The cell migration of U87. **(B)** Quantitative results of U87 cell migration. **(C)** The cell migration of U251. **(D)** Quantitative results of U251 cell migration. Values are the mean of triplicate samples from a representative experiment. *, *P* < 0.05. **, *P* < 0.01.

To further explore the role of GSK-J4 on cell invasion, the cell transwell assay was carried out. The capacity of cell invasion was obviously reduced in glioma cells U87 and U251 after GSK-J4 treatment (Figure 7A and 7C). There is significant difference between GSK-J4 treatment group and control group (*P* < 0.05, Figure 7B and 7D). These results implied that GSK-4 also has a potential on inhibition of glioma metastasis.

## DISCUSSION

There is increasing evidence that histone modifications play an important role in the cancer development [13]. JMJD3, also known as lysine (K)-specific demethylase 6B (KDM6B), is a histone H3K27 demethylase and plays an important role in many processes including tissue regeneration, inflammation, cellular senescence and aging [14, 15]. Abnormal expression or activity of JMJD3 can lead many cancers, such as kidney cancer, breast cancer and glioma [16–18]. Several histone demethylases including JMJD3 have been considered as therapeutic targets for cancer [19, 20].

GSK-J4 is a specific H3K27 demethylase inhibitor, and can increase H3K27me2/3 level and inhibit target genes expression through inhibiting JMJD3 activity [21]. GSK-J4, as a JMJD3 inhibitor, is mainly used in two aspects, immune disease [22] and cancer [23], which originate from the JMJD3 played key role in the two processes [24]. Although GSK-J4 can also be used as a UTX inhibitor of H3K27 demethylation for therapy against T-cell acute lymphoblastic leukemia [25], however, GSK-J4 is used as a JMJD3 inhibitor in most cases [26]. In addition to pediatric glioma [10, 11], GSK-J4 has also showed significant anti-tumor effect on many cancers, such as acute lymphoblastic leukaemia, ovarian cancer and non-small cell lung cancer [27–29].

In this study, we demonstrated that there are increased JMJD3 mRNA expressions in glioblastoma tissues. We also found that many glioma cells also have more JMJD3 content and less H3K27me2/3 level compared to endothelial cells. The result indicates that JMJD3 overexpression is a common phenomenon in glioma tissues and cell lines, which implies GSK-J4 has potential pharmacological effects on them.

Our data also showed that GSK-J4 can selectively inhibit cell proliferation and migration of glioma cell U87 and U251, and specifically induce cell apoptosis. These results provide further evidence that GSK-J4 has also anti-tumor effect for JMJD3-overexpressed glioma, not limited to K27M-mutation pediatric glioma. Therefore, overexpression of JMJD3 can be used as a standard for glioma treatment with GSK-J4. Preview study has demonstrated that GSK-J4-mediated inhibition of JMJD3 can sensitize germinal center B-cell diffuse large B-cell lymphoma cells to chemotherapy agents [30]. And, our results showed that GSK-J4 has only modest growth-inhibitory and cell death-inducing effects on glioma cells. Therefore, we hypothesized that GSK-J4 is more likely to serve as an adjuvant drug and to exert pharmacological effects by increasing chemo-sensitivity. Our preliminary data support the hypothesis. However, further researches are needed to confirm it.

In conclusion, our results showed that GSK-J4 has the modest anticancer activity against JMJD3-overexpressed glioma cells. It also indicated that there is no obvious inhibitory effect of GSK-J4 on endothelial cell, which implies that GSK-J4 has lower side effects. These promising *in vitro* experiments mean that the importance of further animal experiments to evaluate the pharmacological and toxicological effects of GSK-J4 in glioma treatment.

## MATERIALS AND METHODS

### Cell culture and transfection

The human glioma cell lines U87, U251 and brain microvascular endothelial cell (hCMEC) were purchased from China Infrastructure of Cell Line Resources. These cells were maintained in DMEM (GIBCO, Grand Island, USA) supplemented with 10% heated-inactivated fetal bovine serum (FBS, Hyclone, Logan, USA). Cells were cultured at 37°C with 5% CO_2_.

### Cell Counting Kit-8(CCK-8) assay

The pharmacological effects of GSK-J4 (Selleck, Shanghai, China) on cell proliferation were examined by Cell Counting Kit-8 (Dojindo, Kumamoto, Japan) according to the manufacturer's instructions. U87, U251 and hCMEC cells were seeded on the 96-well plates (1 × 10^4^/well) for 24h, then treated with different concentrations of GSK-J4 (0, 0.5, 1, 2, 4 and 8μM) for 48h or treated with 4μM GSK-J4 for different time (24h, 48h and 72h). Cells were added 15 μl of CCK-8 for each well of a 96-well plate and the culture was expanded for 1h. Absorbance was measured at a wavelength of 450 nm using an ELISA microplate reader. Assays were repeated at least three times.

### Flow cytometry analysis

Cell apoptosis was detected using a FITC Annexin V Apoptosis Detection Kit I (BD biosciences, Franklin Lakes, NJ, USA) according to the manufacturer's protocols. Cells were seeded in 6-well plates (1 × 10^6^/well) and treated with 4μM or 8μM GSK-J4 for 48h. Then, cells were harvested, centrifuged, and washed twice with cold PBS. Cells were re-suspended in 1X Annexin V Binding Buffer at a concentration of 1 × 10^6^ cells/ml. 100 μl of the solution (1 × 10^5^ cells) was transferred to a 5ml culture tube, and added 5 μl of FITC Annexin V and 5 μl PI. The cells were gently vortexed and incubated for 15 min at room temperature (25°C) in the dark. Then, each tube was added 400 μl of 1X Annexin V Binding Buffer. Cell apoptosis assay was performed on a flow cytometry (Epics CL, xL, Beckman, CA, USA). These experiments were repeated at least three times.

### Cell migration assay (cell scratch test)

The cells were seeded on the 6-well plates and treated with 8μM GSK-J4 for 24h. Before treatment, a clean line was created with a sterile 200μl pipette tip. The migration of cells was monitored using a digital camera system and imaged at the time of 0h and 24h. The relative cell migration rate was calculated. These experiments were repeated at least three times.

### Quantitative real-time polymerase chain reaction (qRT-PCR)

Total RNAs were extracted from cells using Trizol reagent (Invitrogen, Carlsbad, CA, USA) in accordance with manufacturer's instructions. Then, cDNA was synthesized using a Fermentas RT system (Thermo Scientific, Wilmington, DE, USA) and subjected to qRT-PCR using SYBR Premix Ex Taq™ I (TaKaRa, Otsu, Shiga, Japan) according to the manufacturer's protocol. The primers were synthesized by Sangon (Shanghai, China) and sequences were as follows. JMJD3 primers forward: 5’-CACCCCAGCAAACCATATTATGC-3’; reverse: 3’- CACACAGCCATGCA GGG ATT-5’.β-ACTIN primers forward: 5’-CCACTGGCATCGTGATGGACTCC-3’; reverse: 5’-GCCGTGGTGGTGAAGCTGTAGC-3’. Relative expression level of JMJD3 was normalized to the internal referenceβ-ACTIN.

### Western blots

The cells were washed twice with PBS buffer and homogenized in 200μl radioimmuno-precipitation assay (RIPA) buffer containing the protease inhibitors cocktail(1 mmol/L) and phenylmethylsulfonyl fluoride (100μg/mL). Homogenates were centrifuged and supernatants were collected. A total of 50 μg of protein separated by 10% sodiumdodecyl sulfate-polyacrylamide gel electrophoresis (SDS-PAGE) and transferred to polyvinylidene difluoride (PVDF) membranes. The membranes were saturated with 5% skim milk in TBST (50 mM Tris–HCl, 150 mM NaCl, 0.1% Tween-20) for 2h and then incubated with primary antibodies at 4°C overnight. The primary antibodies used in this study included rabbit polyclonal antibodies to JMJD3 (1:1500, Abcam, Shanghai, China), H3K27me3 (1: 1,500, Cell Signaling Technology, Massachusetts, USA) and H3 (1:2,000, Cell Signaling Technology). The membranes were incubated with HRP-conjugated goat anti-rabbit antibody (1:5,000, Sigma, St Louis, USA) for 1 h at room temperature and exposed to enhanced chemiluminescence substrate (Millipore, Rockford, USA). The detection was performed using a film.

### Statistical analysis

All experimental data were presented as means ±standard deviation (SD) from three independent experiments. The differences between two groups were analyzed using unpaired two-tailed Student *t* test. *P*<0.05 was considered to be statistically significant.

## Abbreviations

JMJD3: jumonji domain-containing protein 3
KDM6B: lysine-specific demethylase 6B
CCK-8: Cell Counting Kit-8
K27M: lysine (K) 27 to methionine (M) mutation
